# Reflections on Criminal Justice Reform: Challenges and Opportunities

**DOI:** 10.1007/s12103-022-09713-5

**Published:** 2022-12-17

**Authors:** Pamela K. Lattimore

**Affiliations:** RTI International, 3040 East Cornwallis Road, Research Triangle Park, NC 27703 USA

**Keywords:** Criminal Justice Reform, War on Drugs, War on Crime, US Correctional

## Abstract

Considerable efforts and resources have been expended to enact reforms to the criminal justice system over the last five decades. Concerns about dramatic increases in violent crime beginning in the late Sixties and accelerating into the 1980s led to the “War on Drugs” and the “War on Crime” that included implementation of more punitive policies and dramatic increases in incarceration and community supervision. More recent reform efforts have focused on strategies to reduce the negative impacts of policing, the disparate impacts of pretrial practices, and better strategies for reducing criminal behavior. Renewed interest in strategies and interventions to reduce criminal behavior has coincided with a focus on identifying “what works.” Recent increases in violence have shifted the national dialog from a focus on progressive reforms to reduce reliance on punitive measures and the disparate impact of the legal system on some groups to a focus on increased investment in “tough on crime” criminal justice approaches. This essay offers some reflections on the “Waged Wars” and the efforts to identify “What Works” based on nearly 40 years of work evaluating criminal justice reform efforts.

The last fifty-plus years have seen considerable efforts and resources expended to enact reforms to the criminal justice system. Some of the earliest reforms of this era were driven by dramatic increases in violence leading to more punitive policies. More recently, reform efforts have focused on strategies to reduce the negative impacts of policing, the disparate impacts of pretrial practices, and better strategies for reducing criminal behavior. Renewed interest in strategies and interventions to reduce criminal behavior has coincided with a focus on identifying “what works.” Recent increases in violence have shifted the national dialog about reform. The shift may be due to the disruptions caused by the COVID-19 epidemic or concerns about the United States returning to the escalating rise in violence and homicide in the 1980s and 1990s. Whichever proves true, the current rise of violence, at a minimum, has changed the tenor of policymaker discussions, from a focus on progressive reforms to reduce reliance on punitive measures and the disparate impact of the legal system on some groups to a focus on increased investment in “tough on crime” criminal justice approaches.

It is, then, an interesting time for those concerned about justice in America. Countervailing forces are at play that have generated a consistent call for reform, but with profound differences in views about what reform should entail. The impetus for reform is myriad: Concerns about the deaths of Black Americans by law enforcement agencies and officers who may employ excessive use of force with minorities; pressures to reduce pretrial incarceration that results in crowded jails and detention of those who have not been found guilty; prison incarcerations rates that remain the highest in the Western world; millions of individuals who live under community supervision and the burden of fees and fines that they will never be able to pay; and, in the aftermath of the worst pandemic in more than a century, increasing violence, particularly homicides and gun violence. This last change has led to fear and demands for action from communities under threat, but it exists alongside of other changes that point to the need for progressive changes rather than reversion to, or greater investment in, get-tough policies.

How did we get here? What have we learned from more than 50 years of efforts at reform? How can we do better? In this essay, I offer some reflections based on my nearly 40 years of evaluating criminal justice reform efforts.^1^

## Part I: Waging “War”

The landscape of criminal justice reform sits at the intersection of criminal behavior and legal system response. Perceptions of crime drive policy responses. Perceptions of those responsible for crime also drive responses. And perceptions of those responses result in demands for change. To establish context for the observations that follow, this section describes trends in crime, the population of justice-involved individuals, and the expenditures supporting the sprawling criminal justice enterprise in the United States since the mid-to-late twentieth century.

But first, my perspective: Over the last nearly 40 years, I have observed justice system reform efforts since working, while a first-year graduate student in 1983, on a National Institute of Justice (NIJ) grant that funded a randomized control trial of what would now be termed a reentry program (Lattimore et al., 1990). After graduate school, I spent 10 years at NIJ, where I was exposed to policy making and the relevance of research for both policy and practice. I taught for several years at a university. And, for most of my career, I have been in the trenches at a not-for-profit social science research firm. Throughout my career, I have conducted research and evaluation on a broad array of topics and have spent most of my time contemplating the challenges of reform. I’ve evaluated single programs, large federal initiatives, and efforts by philanthropies to effect reform. I’ve used administrative data to model criminal recidivism to address—to the degree statistical methods allow—various dimensions of recidivism (type, frequency, and seriousness). I’ve developed recidivism models for the practical purpose of assessing risk for those on community supervision and to explore the effects of covariates and interventions on recidivism and other outcomes. I’ve participated in research attempting to understand the shortcomings of and potential biases in justice data and the models that must necessarily use those data. While most of my work has focused on community corrections (e.g., probation and post-release interventions and behavior) and reentry, I have studied jail diversion programs, jail and pretrial reform, and efforts focused on criminal record expungement. These experiences have illuminated for me that punitiveness is built into the American criminal justice system—a punitiveness that traps many people from the time they are first arrested until they die.

## Crime and Correctional Population Trends

**Fig. 1:**
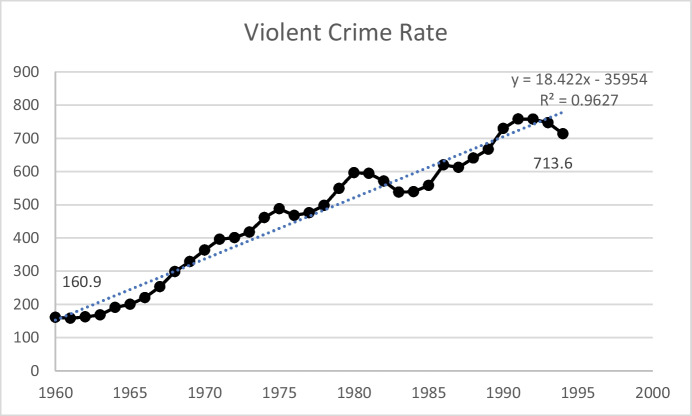
US Violent Crime Rate, 1960–1994

The 1960s witnessed a dramatic rise in crime in the United States, and led to the so-called “War on Crime,” the “War on Drugs,” and a variety of policy responses, culminating with the passage of the Violent Offender Incarceration and Truth-in-Sentencing Act of 1994 (“The 1994 Crime Act”; Pub. L. 103–322). Figure 1 shows the violent crime rate in the United States from 1960 to 1994.^2^ In 1960, the violent crime rate in the United States was 161 per 100,000 people; by 1994 the rate had increased more than four-fold to nearly 714 per 100,000.^3^ As can be seen, the linear trend was highly explanatory (R-square = 0.96)—however, there were two obvious downturns in the trend line—between 1980 and 1985 and, perhaps, between 1991 and 1994.

**Fig. 2:**
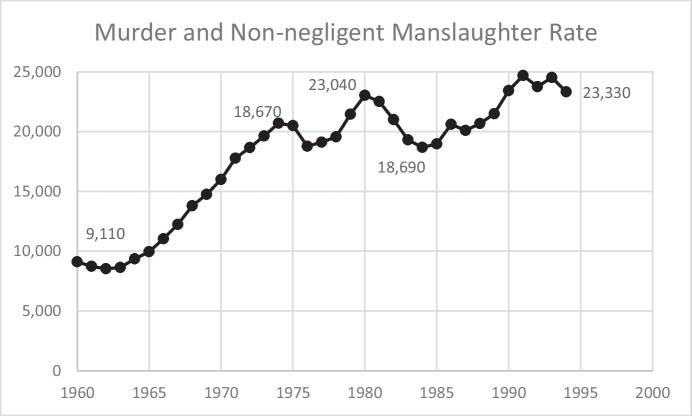
United States Murder and Non-negligent Manslaughter Rate, 1960–1994

Homicides followed a similar pattern. Figure 2 shows the number of homicides each year between 1960 and 1994. In 12 years (1960 to 1972), the number of homicides doubled from 9,100 to 18,670. By 1994, the number had grown to 23,330—but it is worth noting that there were multiple downturns over this period, including a drop of more than 4,000 between 1980 and 1984. These figures show the backdrop to the “War on Drugs” and the “War on Crime” that led reformers to call for more punitive sentencing, including mandatory minimum sentences, “three-strikes laws” that mandated long sentences for repeat offenders, and truth-in-sentencing statutes that required individuals to serve most of their sentences before being eligible for release. This was also the period when the 1966 Bail Reform Act, which sought to reduce pretrial detention through the offer of money bond, was supplanted in 1984 by the Pretrial Reform Act, which once again led to increased reliance on pretrial detention.

The 1994 Crime Act, enacted during the Clinton Administration, continued the tough-on-crime era by enabling more incarceration and longer periods of incarceration that resulted in large increases in correctional populations. In particular, the Violent Offender Incarceration and Truth-in-Sentencing (VOI/TIS) Incentive Grant Program, funded by the Act, provided $3 billion to states to expand their jail and prisons capacities between FY1996 and FY2001 and to encourage states to eliminate indeterminate sentencing in favor of laws that required individuals to serve at least 85% of their imposed sentences.

**Fig. 3:**
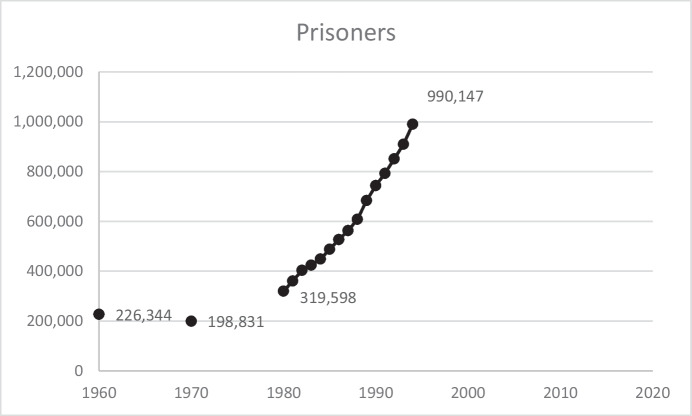
State and Federal Prisoners in the US, 1960–1994

Figure 3 shows the dramatic rise in the number of state and federal prisoners *prior* to passage of the 1994 Crime Act—the number of prisoners more than tripled between 1980 and 1994.^4^ The increase in numbers of prisoners was not due to shifts from jail to prison or from probation to prison, given that all correctional populations increased dramatically over this 14-year period—jail populations increased 164% (183,988 to 486,474), probation increased 166% (1,118,097 to 2,981,022), and parole increased 213% (220,438 to 690,371).

**Fig. 4:**
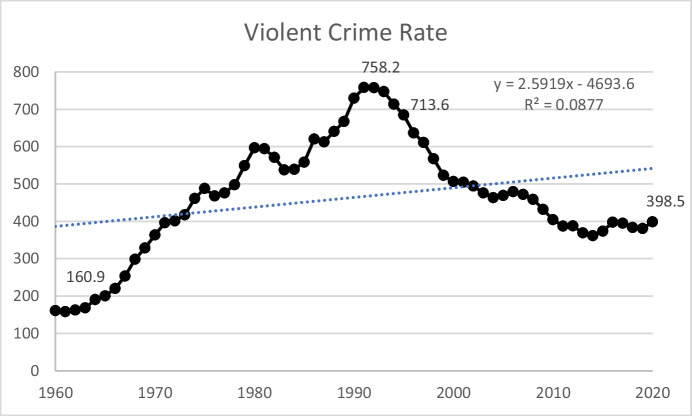
United States Violent Crime Rate (violent crimes per 100,000 population), 1960–2020

So, what happened *after* passage of the 1994 Crime Act? Fig. 4 shows the violent crime rate from 1960 through 2020. As can be seen, the *decrease* in the violent crime rate that began *prior* to passage of the 1994 Crime Act continued. And, notably, it preceded the influx of federal funding to put more police on the streets, build more jails and prisons, and place more individuals into the custody of local, state, and federal correctional agencies. Even with a small increase between 2019 and 2020, the violent crime rate in 2020 was 398.5 per 100,000 individuals, well below its 1991 peak of 758.2.^5^

**Fig. 5:**
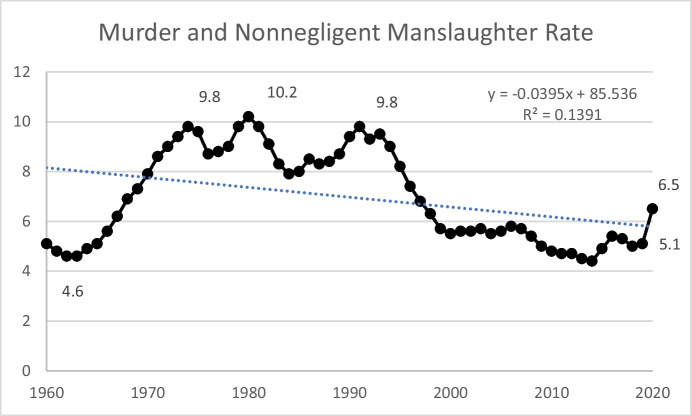
United States Murder and Nonnegligent Manslaughter Rate, 1960–2020

Figure 5 shows the US homicide rate from 1960 to 2020. Consistent with the overall violent crime rate, the homicide rate in 2020 remained well below the peak of 10.2 that occurred in 1981. (Rates also may have risen in 2021—as evidenced by reports of large increases in major U.S. cities—but an official report of the 2021 number and rate for the U.S. was not available as of the time of this writing.) The rise in this rate from 2019 to 2020 was more than 27%— worthy of attention and concern. It represents the largest year-over-year increase between 1960 and 2020. However, there have been other years where the rate increased about 10% (1966, 1967, 1968, 2015, and 2016), only then to drop back in subsequent years. Further, it is difficult to determine whether the COVID-19 pandemic, which has caused massive disruptions, is a factor in the increase in homicides or to know whether the homicide rate will abate as the pandemic ebbs. Finally, it bears emphasizing that during this 60-year period there have been years when the homicide rate fell by nearly 10% (e.g., 1996, 1999). From a policy perspective, it seems prudent to be responsive to increases in crime but also not to over-react to one or two years of data—particularly during times of considerable upheaval.

**Fig. 6:**
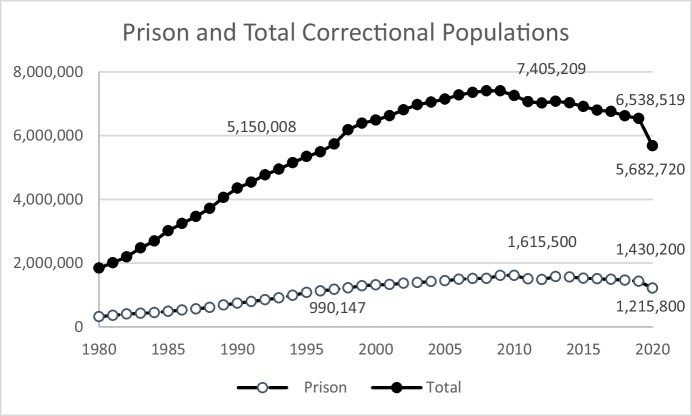
United States Prison and Total Correctional Populations, 1980–2020

**Fig. 7:**
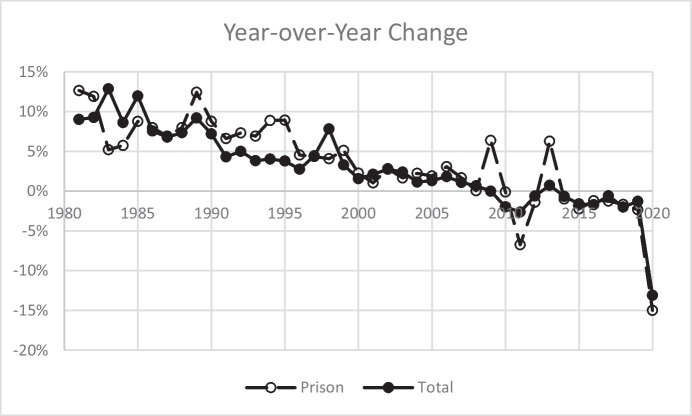
Year-over-Year Change in Prison and Total Correctional Populations, 2981–2020

The growth in correctional populations, including prisoners, that began in the 1970s continued well into the twenty-first century—in other words, long after the crime rate began to abate in 1992. Figure 6 shows the prison population and total correctional population (state and federal prison plus jail, probation, and parole populations summed) between 1980 and 2020. Both trends peaked in 2009 at 1,615,500 prisoners and 7,405,209 incarcerated or on supervision. Year-over-year decreases, however, have been modest (Fig. 7), averaging about 1% (ignoring the steep decline between 2019 and 2020). The impact of factors associated with COVID-19, including policy and practice responses, resulted in a 15% decrease in the numbers of state and federal prisoners and a 14% decrease in the total number of adults under correctional control. Based on ongoing projects in pretrial and probation, as well as anecdotal evidence related to court closures and subsequent backlogs, it is reasonable to assume that some, if not most, of the decline in populations in 2020 was due to releases that exceeded new admissions as individuals completed their sentences and delays in court processing reduced new admissions. To the extent that these factors played a role, it is likely that in the immediate near term, we will see numbers rebound to values closer to what prevailed in 2019.

## Responding with Toughness (and Dollars)

The increase in crime beginning in the 1960s led to a political demand for a punitive response emphasized by Richard Nixon’s “War on Crime” and “War on Drugs.” In 1970, Congress passed four anticrime bills that revised Federal drug laws and penalties, addressed evidence gathering against organized crime, authorized preventive detention and “no-knock” warrants, and provided $3.5 billion to state and local law enforcement.^6^ Subsequent administrations continued these efforts, punctuated by the Crime Act of 1994. As described by the U.S. Department of Justice:

The Violent Crime Control and Law Enforcement Act of 1994 … is the largest crime bill in the history of the country and will provide for 100,000 new police officers, $9.7 billion in funding for prisons and $6.1 billion in funding for prevention programs …. The Crime Bill provides $2.6 billion in additional funding for the FBI, DEA, INS, United States Attorneys, and other Justice Department components, as well as the Federal courts and the Treasury Department.^7^

Much of the funding went to state and local agencies to encourage the adoption of mandatory minimum sentences, “three strikes” laws, and to hire 100,000 police officers and build prisons and jails. This funding was intended to steer the highly decentralized United States criminal justice “system” towards a more punitive approach to crime; this system encompasses all levels of government (local, state, and federal) and all branches of government (executive, judicial, legislative).

**Fig. 8:**
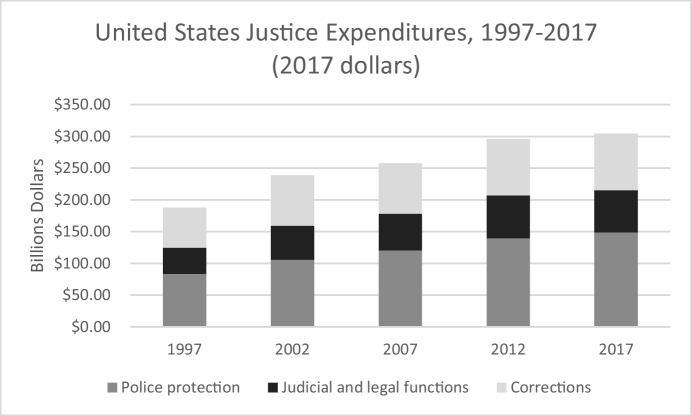
United States Justice Expenditures, 1997–2017

The nation’s crime rate peaked in 1992. So, this “largest crime bill in the history of the country” began a dramatic increase in funding for justice expenditures just as crime had already begun to decline. Figure 8 shows that expenditures increased roughly 50% in real dollars between 1997 and 2017—from $188 billion to more than $300 billion dollars (Buehler, 2021).^8^ More than half of that increase-—$65.4 billion additional—went to police protection. Roughly $50 billion additional went to the judiciary and corrections.

**Fig. 9:**
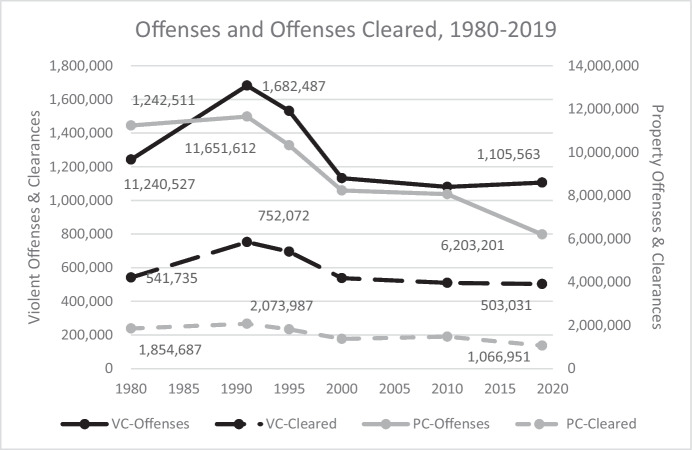
Offenses Known and Cleared in the US, Selected Years 1980–2019

So, what did these increases buy? Dramatically declining crime rates (Figs. 4 and 5) suggest that numbers of crimes also declined. That can be seen in Fig. 9, which shows offenses known and an estimate of offenses cleared for selected years between 1980 and 2019.^9^ In 1991, there were 11,651,612 known property offenses and 1,682,487 known violent offenses—these numbers declined 47% and 34% by 2019.

Declining numbers of crimes and dramatic increases in expenditures on policing and justice system operation would suggest that there should have been improvements in offense clearance rates during this time. This did not happen. Crime clearance rates stayed roughly constant, which means that the numbers of offenses cleared declined by percentages like declines in the number of offenses over this period—49% for property offenses and 33% for violent offenses. More than 750,000 violent offenses and more than 2 million property offenses were cleared in 1991 compared to about 500,000 violent offenses and 1 million property offenses in 2019.

Presently, as violent crime ticks up, we are hearing renewed calls for “tough-on-crime” measures. Some opinion writers have compared 2022 to Nixon’s era. Kevin Boyle noted:

[Nixon] already had his core message set in the early days of his 1968 campaign. In a February speech in New Hampshire, he said: “When a nation with the greatest tradition of the rule of law is torn apart by lawlessness, when a nation which has been the symbol of equality of opportunity is torn apart by racial strife … then I say it’s time for new leadership in the United States of America.” There it is: the fusion of crime, race and fear that Nixon believed would carry him to the presidency.^10^

Responding to the recent increase in violent crime, President Joseph Biden proposed the Safer America Plan to provide $37 billion “to support law enforcement and crime prevention.”^11^ The Plan includes more than $12 billion in funds for 100,000 additional police officers through the Community Oriented Policing Services (COPS) program. This proposal echoes the “100,000 cops on the street” that was a centerpiece of the 1994 Crime Act, which created the COPS office and program. Unlike the 1994 Crime Act, however, the Safer America Plan does not include funding for prisons and jails. Both the 1994 Crime Act and the Safer America Plan address gun violence, strengthen penalties for drug offenses, and provide support for programs and interventions to make communities safer and to address criminal recidivism.

The previous 50 or 60 years witnessed reforms efforts other than these that largely focused on bolstering the justice system infrastructure. The 1966 Bail Reform Act sought to reduce pretrial detention through the offer of money bond, but subsequently was supplanted by the 1984 Pretrial Reform Act that once again promoted pretrial detention.^12^ This century—as jail populations exceeded 700,000, with most held prior to conviction—there has been considerable attention to eliminate money bond, which disproportionately leads to pretrial detention for poor and marginalized individuals (and release for the “well-heeled”). Private philanthropy has led much of this focus on pretrial and bail reform. For example, the MacArthur Foundation has spent several hundred million dollars on their Safety and Justice Challenge since 2015 with a goal of reducing jail populations and eliminating racial and ethnic disparity.^13^ The Laura and John Arnold Foundation (LJAF) took a different approach and has invested substantial sums in the development and validation of a pretrial assessment instrument (the Public Safety Assessment or PSA) that provides assessments of the likelihood an individual will fail to appear to court or be arrested for a new crime or new violent crime if released while awaiting trial.^14^ Although assessment algorithms have been criticized for lack of transparency and for perpetuating racial bias, the PSA scoring algorithm is publicly available and has not shown evidence of racial bias in a series of local validations conducted by RTI for LJAF. New York and New Jersey are among the states that have attempted to reduce reliance on money bond. However, as violent crime has increased, these efforts have faced considerable pushback.

The bail bonds industry has been a vocal opponent of efforts to reduce or eliminate the use of money bond. This industry is not the only one that profits from the imposition of punishment. As Page and Soss (2021) recently reported, “Over the past 35 years, public and private actors have turned US criminal justice institutions into a vast network of revenue-generating operations. Today, practices such as fines, fees, forfeitures, prison charges, and bail premiums transfer billions of dollars from oppressed communities to governments and corporations.” Fines, fees, and forfeitures generally profit the governments and agencies that impose them—although supervision fees to private probation services benefit businesses, as do fees for electronic monitoring, and drug testing. The Prison Policy Institute reports that there are more than 4,000 companies that profit from mass incarceration.^15^ Court and supervision fees can quickly add up to hundreds or even thousands of dollars, burdening people with crushing debt and the threat of jail if they don’t pay.^16^ There can be other consequences as well. After Florida passed a constitutional amendment to restore voting rights to individuals once they had completed their carceral or community sentence, the State specified that the right to vote would not be restored until an individual had paid all outstanding fees and fines. In addition, mistakenly voting with outstanding fees and fines is a felony.^17^

Other work to reform pretrial justice includes early provision of defense counsel, and implementation of diversion programs for individuals charged with low-level offenses or who have behavioral health issues. The sixth amendment to the United States Constitution guarantees criminal defendants in the United States a right to counsel. In some jurisdictions (and the Federal court system), this is the responsibility of an office of public defense. In others, private defense counsel is appointed by the Court. Regardless, public defense is widely understood to be poorly funded. As noted by Arnold Ventures, a philanthropy currently working to improve access to defense, “The resulting system is fragmented and underfunded; lacks quality control and oversight; and fails to safeguard the rights of the vast majority of people charged with crimes who are represented by public defenders or indigent counsel.”^18^

Mental health problems are prevalent among individuals incarcerated in local jails and prisons. The Bureau of Justice Statistics, in a report by Bronson and Berzofsky (2017), reported that “prisoners and jail inmates were three to five times as likely to have met the threshold for SPD [serious psychological distress] as adults in the general U.S. population.” Bronson and Berzofsky further reported that 44% of individuals in jail reported being told they had a mental disorder. The Substance Abuse and Mental Health Administration’s GAINS Center has been at the forefront of efforts to implement jail diversion programs for individuals with mental health or substance use disorders and has also played a significant role in the establishment of treatment courts.^19^ Crisis Intervention Training (CIT) for law enforcement to improve interaction outcomes between law enforcement and individuals in crisis. The National Alliance on Mental Illness (NAMI) notes that “The lack of mental health crisis services across the U.S. has resulted in law enforcement officers serving as first responders to most crises. A Crisis Intervention Team (CIT) program is an innovative, community-based approach to improve the outcomes of these encounters.”^20^ Non-law enforcement responses—such as the CAHOOTS program that was developed in Eugene, Oregon—to certain calls for service are also being tested in multiple communities.^21^ Despite multiple efforts to identify appropriate alternatives to jail, individuals with mental health disorders continue to disproportionately fill the nation’s jails.

## A Recapitulation

The 1970 crime bills that passed early in Nixon’s presidency set the stage for the infusion of federal dollars that has provided billions of dollars in funding for police and prisons. Between 1970 and 1994, the number of adults in state and federal prisons in the United States increased from less than 200,000 to nearly 1 million. In 2019, that number stood at more than 1.4 million down from its peak in 2009. Another 734,500 individuals were in jail and more than 4.3 million were in the community on probation or parole. Although representing a dramatic decline since these populations peaked about 2009, this still means that more than 6 million adults were under the supervision of federal, state, and local corrections agencies in 2019.

Thus, it is important to recognize that we are at a very different place from the Nixon era. Today, the numbers (and rates) of individuals who are “justice-involved” remain at near record highs. As the progressive efforts of the twenty-first century encounter headwinds, it is worth waving a caution flag as the “remedies” of the twentieth century—more police, “stop and frisk,” increased pretrial detention—are once again being proposed to address violent crime.

## Part II: Finding “What Works”

The 1994 Crime Act and subsequent reauthorizations also included funding for a variety of programs, including drug courts, prison drug treatment programs, and other programs focused on facilitating reentry and reducing criminal recidivism. Subsequent legislation authorized other Federal investments that resurrected rehabilitation as a goal of correctional policy. The Serious and Violent Offender Reentry Initiative (SVORI) provided $100 million (and some limited supplements) to agencies to develop programs that began in prison and continued into the community and were intended to improve outcomes across a range of domains—community reintegration, employment, family, health (including mental health), housing, substance abuse, supervision compliance and, of course, recidivism (see Lattimore et al., 2005b; Winterfield et al., 2006; Lattimore & Visher, 2013, 2021; Visher et al., 2017). Congress did not reauthorize SVORI but instead authorized the Prisoner Reentry Initiative (PRI) managed by the U.S. Department of Labor; PRI (now the Reintegration of Ex-Offenders or RExO program) provides funding for employment-focused programs for non-violent offenders. In 2006, a third reentry-focused initiative was funded—the Marriage and Incarceration Initiative was managed by the Department of Health and Human Services and focused on strengthening marriage and families for male correctional populations. In 2008, Congress passed the Second Chance Act (SCA) to provide grants for prison and jail reentry programs. The SCA grant program administered by the Bureau of Justice Assistance (BJA) was reauthorized in 2018; it continues to provide reentry grants to state and local agencies (see Lindquist et al., 2021). These initiatives all primarily focused on supporting efforts at the state and local level. The First Step Act of 2018 focused on reforms for the federal prison system. These efforts signified a substantial increase in efforts aimed at determining “what works” to reduce criminal behavior—and provided an opportunity to rebut the “nothing works” in correctional programming that followed the publication of research by Lipton (1975).

Elsewhere, I have summarized some of the research into Federal initiatives that I have conducted over the years (Lattimore, 2020). These studies comprise work in dozens of states, involving thousands of individuals and have included studies of drug treatment, jail diversion, jail and prison reentry, and probation. Some involved evaluation of a substantial Federal investment, such as the multi-site evaluation of SVORI.

These evaluations, as has been largely true of those conducted by others, have produced mixed results. Systematic reviews and meta-analyses focusing on the effectiveness of adult correctional programming have yielded findings of modest or negligible effects (e.g., Aos et al., 2006; Bitney et al., 2017; Lipsey & Cullen, 2007; MacKenzie, 2006; Sherman, et al., 1997). In an updated inventory of research- and evidence-based adult programming, the Washington State Institute for Public Policy (Wanner, 2018) identified a variety of programs for which evidence suggests significant if modest effect sizes. As has been identified by others (e.g., MacKenzie, 2006), the most effective programs focused on individual change, including, for example, cognitive behavior therapy (estimated effect size of -0.11). Treatment-oriented intensive supervision programs were found to reduce recidivism by about 15%, while surveillance-oriented intensive supervision was found to have no demonstrated effects. Several types of work and educational programs (correctional industries, basic adult education, prison-based vocational education, and job training and assistance in the community) were found to reduce recidivism between 5 and 22%. Most non-zero treatment effect sizes were between about 5% and 15%. Lipsey and Cullen (2007) also suggest 14% to 22% reductions in recidivism for adult rehabilitation treatment programs.

Two thoughts about these small effects warrant consideration. The first, of course, is why reducing criminal behavior appears to be so difficult. Second, however, is that, in recognizing the first, perhaps we should adapt more realistic expectations about what can be achieved and acknowledge that even small effects can have a meaningful impact on public safety.

## Challenges: Why Is Effective Criminal Justice Reform So Difficult?

One issue with most federal funding streams is “short timelines.” For example, typical of grant programs of this type, SVORI grantees were given *three* years of funding. During this time, they had to develop a programmatic strategy, establish interagency working arrangements, identify program and service providers, develop a strategy for identifying potential participants, and implement their programs. Three years is a very short time to *develop* a program that incorporates needs assessment, provides a multiplicity of services and programs within an institution, *and* creates a path for continuation of services as individuals are released to various communities across a state.

The “short timelines” problem underlies, and contributes to, a variety of other considerations that can plague efforts to identify “what works.” Based on my experiences, these considerations, which I discuss further below, include the following:

1. People: Justice-involved individuals have multiple needs and there is an emerging question as to whether addressing these needs is the best path to desistance.
2. Programs: Interventions often lack adequate logic models and are poorly implemented.
3. Methods: Evaluations frequently are underpowered and unlikely to scale the alpha 0.05 hurdle typically used to identify statistically significant effects.

### People

First, it is important to recognize that justice-involved individuals face serious and complex challenges that are difficult to remedy. Many scholars have highlighted the myriad of challenges faced by individuals returning to the community from prison (e.g., see Petersilia, 2003; Travis, 2005; Travis & Visher, 2005). In interviews conducted with 1,697 men and 357 women who participated in the SVORI multisite evaluation, 95% of women and 94% of men said at the time of prison release that they needed more education. Nearly as many—86% of women and 82% of men—said they needed job training. More than two-thirds indicated that they needed help with their criminal thinking and three-quarters said they needed life skills training. They were somewhat less likely to report needing substance use disorder or mental health treatment but still—at the time of prison release—66% of the women and 37% of the men reported needing substance use treatment and 55% of the women and 22% of the men reported needing mental health treatment.

**Fig. 10:**
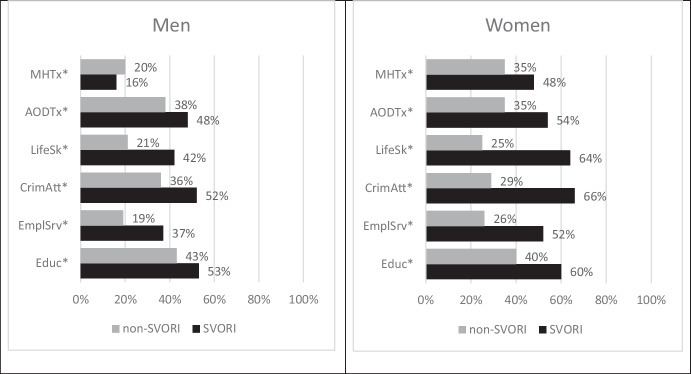
Self-reported service receipt during incarceration for SVORI program evaluation participants. Note: * = *p* <  = 0.05. Educ = educational programming, EmplSrv = employment-related services, CrimAtt = programs for criminal attitudes including cognitive behavior therapy, LifeSk = life skills, AODTx = substance abuse treatment, and MHTx = mental health treatment. Sample sizes were SVORI men (863), non-SVORI men (834), SVORI women (153) and non-SVORI women (204). Source: Lattimore & Visher (2009)

Half of these individuals had participated in SVORI programs while incarcerated and the proportions reported reflect their self-assessment of need *after* in-prison receipt of programming. Figure 10 shows the percentages of SVORI and non-SVORI groups who reported receiving a select set of services and programs during their incarceration. Several things standout: (1) The receipt of programs and services during incarceration was much less than the indicated need at the time of release; and (2) SVORI program participants were more likely to report receiving services than the comparison group members who were not in SVORI programs.

More recently, Lindquist et al. (2021) completed a seven-site evaluation of Second Chance Act reentry programs that were a mix of jail- and prison-based programs. About half of the study participants reported having received substance use disorder treatment and about one-third reported having received mental health treatment. At release, they reported limited-service receipt. For example, there was no significant difference between receipt of educational programming (23% of SCA program participants compared with 17% for comparison group members). SCA program participants were more likely to report receiving any employment services (60% versus 40%), which included job assistance, employment preparation, trade or job training programs, vocational or technical certifications, and transitional job placement or subsidized employment. SCA program participants were also more likely to report receiving cognitive behavioral services (58% versus 41%). But, again, not all program participants received services despite needing them and some comparison subjects received services.

Limited access to treatment by program participants and some access to treatment by comparison subjects were also observed in a multi-site study of pre- and post-booking jail diversion programs for individuals with co-occurring substance use disorder and serious mental illness (Broner et al., 2004; Lattimore et al., 2003). Across eight study sites, 971 diverted subjects and 995 non-diverted subjects were included in this evaluation; the research found only modest differences in the receipt of services and treatment at 3- and 12-months follow-up. For example, at the 3-month interview, 26% of both groups reported receiving substance abuse counseling, and at the 12-month interview, 0.7% of those diverted versus no non-diverted participant received two or more substance abuse counseling sessions. At 3 months, 38% of the diverted subjects and 30% of the non-diverted reported mental health counseling versus 41% and 38% at 12 months, respectively.

The service needs expressed by these individuals reflect their lack of education, job experience, vocational skills, and life skills, as well as the substance abuse and mental health issues identified among justice-involved individuals. The intervention response to these needs is reflected in the variety of services prescribed in the typical “reentry program bucket.” These involve the services and programs shown in Fig. 10, as well as case management and reentry planning to coordinate services with respect to needs.

The identification of needs followed by efforts to meet those needs underlies the Risk-Needs-Responsivity (RNR) approach to addressing justice-involved populations (e.g., Andrews & Bonta, 1994, 2006; Latessa, 2020). The RNR approach to addressing criminal behavior is premised on the assumption that if you address identified needs that are correlated with criminal behavior, that behavior will be reduced. In other words, recidivism can be addressed by providing individuals the education and job skills and treatment they need to find gainful employment, reduce substance use, and mitigate symptoms of mental illness. Latessa (2020) recently discussed the RNR approach, reiterating the importance of assessing individual criminogenic and non-criminogenic needs to improve reentry programs. He also reiterated the importance of focusing resources on those identified as high (or higher) risk by actuarial risk assessment instruments—pointing to important work he conducted with colleagues that found that interventions reduced recidivism among high-risk individuals and *increased* it among low-risk individuals (Lowenkamp & Latessa, 2002; Latessa et al., 2010). This approach to reentry programming is reflected in the requirements of most federal grants—like the SVORI and SCA—that require programs to incorporate reentry planning that includes needs assessment and services that address criminogenic and non-criminogenic needs.

As noted, most justice-involved individuals have limited education and few job skills, and many have behavioral health issues, anger management issues, and limited life skills. But if addressing these deficits is the key to successfully rehabilitating large numbers of individuals caught in the carceral and community justice system, the meager results of recent research suggests two possibilities. First, this is the right approach, but poor or incomplete implementation has so far impeded findings of substantial effects (a common conclusion since the Martinson report). Second, alternatively, this approach is wrong (or insufficient), and new thinking about the “what and how” of rehabilitative programming is needed. I address the second idea next and turn to the first idea shortly.

MacKenzie (2006) and others (e.g., Andrews and Bonta, 2006; Andrews et al., 1990; Aos et al., 2006; Lipsey, 1995; Lipsey & Cullen, 2007) have stressed that programs focused on individual change have been found to be effective more often than those providing practical services. The SVORI evaluation also found support for this conclusion. Services we classified as “practical” (e.g., case manager, employment services, life skills, needs assessment, reentry planning, and reentry program) were associated with either no or a deleterious impact on arrest chances—although few were statistically different from a null effect. Individual-change services (e.g., anger management, programs for criminal attitudes including cognitive behavior therapy, education, help with personal relationships, and substance abuse treatment) were associated with positive impacts on arrest. The original SVORI evaluation had a follow-up period of about 2 years and findings suggested that the overall impact of SVORI program participation on rearrest and reincarceration were positive but not statistically significant. In contrast to these findings, a longer follow-up that extended at least 56 months showed participation in SVORI programs was associated with longer times to arrest and fewer arrests after release for both men and women. For the men, SVORI program participation was associated with a longer time to reincarceration and fewer reincarcerations, although the latter result was not statistically significant (*p* = 0.18). For the women, the reincarceration results were mixed and not significant.

Support for positive impacts of programs focused on individual change are consistent with theories associated with identity transformation and desistance from criminal activity. Bushway (2020) has recently discussed two alternative views of desistance, contrasting the implications of desistance either as a process (i.e., the gradual withdrawal from criminal activity) or reflective of an identify shift towards a more prosocial identity. In examining these two ideas, Bushway posits that the second suggests that individuals with a history of a high rate of offending may simply stop (as opposed to reducing the frequency of criminal acts). If individuals do (or can or will) stop, the implication is clear: policies that focus on an individual’s criminal history (e.g., for employment or parole decisions) may fail to recognize that the individual has changed. This change may be evidenced by in-prison good behavior (e.g., completing programs and staying out of trouble) or positive steps following release (e.g., actively seeking meaningful employment or engaging in positive relationships). Tellingly, Bushway (2020) notes: “Individuals involved in crime get information about how they are perceived by others through their involvement in the criminal justice system. Formal labels of ‘criminal’ are assigned and maintained by the criminal justice system. As a result, identity models are much more consistent theoretically with an empirical approach that revolves around measures of criminal justice involvement rather than criminal offending per se.” He goes on to discuss the relationship of identity-based models of stark breaks and criminal career models. In short, reflecting insights that labeling theorists have long emphasized, the labels the criminal justice system and society place on individuals may impede the desistance process that is the supposed goal of the system.

### Programs

The second consideration are concerns about program design and implementation—What is the underlying logic model or theory of change? Is there adequate time to develop the program and train staff to implement it appropriately? Is the resulting program implemented with fidelity? The two or three years usually provided to implement complex programs suggest that these goals are unlikely to be met. The “notorious” findings of Martinson (1975) that “nothing works” was more appropriately interpreted as “nothing was implemented.” Unfortunately, nearly 50 years later, we largely observe something similar—not “nothing” but “something” that is far short of what was intended.

As discussed in detail by Taxman (2020), the usual approach to program development and testing skips over important formative steps, doesn’t allow time for pilot testing, and provides little opportunity for staff training or for achievement and maintenance of program fidelity (if there is even a program logic model). From an evaluator’s perspective, this short timeline imposes multiple challenges. An evaluator must identify study participants (and control or comparison subjects), follow them largely while they are in the program, and hope to have at least one year of post-program follow-up—generally without being able to accommodate the impact of likely weak implementation on evaluation power to detect effects.

Thus, it may not be surprising that effects are generally small. However, these small effects may not be negligible from a public safety perspective. In a study of the effects of non-residential drug treatment for a cohort of probationers, Lattimore et al. (2005a) found that treatment reduced the number of probationers with a felony arrest by 23% during the first year and 11% over the first two years. The total number of arrests was also reduced by 17% over 12 months and 14% over 24 months. “Back of the envelope” calculations suggested that if treatment cost $1,000 per individual, it would have been cost effective to provide treatment to all members of the cohort as long as the (average) cost of arrest (and all related criminal justice processing and corrections) exceeds about $6,463.

Another example is to consider that the impact of a treatment effect in the 10% range applied across all prison releases would imply the aversion of many crimes. For example, assuming 750,000 prison releases each year over a five-year period and a 66% rearrest rate within 3 years (and no additional arrests after 3 years), then 3.75 million prisoners will be released over the five years; of these individuals, 2.475 million will be arrested *at least once* during the three years following release. A 10% reduction in *first-time rearrests* would mean 247,500 fewer first-time rearrests. To the extent that many offenders are arrested multiple times, this figure represents a lower bound on the number of averted arrests. A similar analysis could be conducted assuming 2,000,000 probation admissions each year and a 39% rearrest rate within 3 years. In this case, there would be 10,000,000 probation admissions that would generate 3.9 million first-time arrests over the three years after admission to probation. A 10% reduction in first-time rearrests would mean 390,000 fewer arrests. In total, therefore, reducing recidivism—as measured by rearrest by 10% for these hypothetical correctional populations—would translate into 637,500 averted arrests. Extrapolating further and assuming that roughly 10% of the arrests were for violent crime and 90% for property crime, and applying the inverse of the crime clearance rates for these two types of crime to generate a “crimes averted” count, we find that a 10% reduction in recidivism for these two populations would translate into 140,110 violent and 3,519,939 property crimes averted.^22^ Thus, “modest” improvements in recidivism may provide substantial public benefits—in crimes averted, and lower demands on law enforcement, prosecution, and correctional resources.^23^

### Methods

The third consideration is the adequacy of the evaluation methods we routinely apply to this complex problem of inadequate interventions that are partially and sometimes poorly implemented. At minimum, we need to explicitly recognize the impacts of the following:

- Programs partially implemented and partially treated control conditions.
- Recidivism outcomes conditioned on an intermediate outcome.
- Follow-up periods too short to accommodate short-term failure followed by long-term success.
- Focusing on a binary indicator of recidivism ignores frequency and seriousness of offending.

The impact of partial treatment of both treatment and control groups on effect sizes and the consequential impact on statistical power is seldom discussed—either in initial estimates of needed sample sizes or in subsequent discussions of findings. As shown earlier and is true for most justice evaluations, the control or comparison condition is almost never “nothing.” Instead, it is generally “business as usual” (BAU) that means whatever the current standard of treatment entails. Thus, the treatment group may receive some services that aren’t available to the control group, but in many cases both groups have access to specific services and programs although the treatment group may get priority.

As we saw in Fig. 10, treatment was reported by some individuals in both the SVORI and non-SVORI groups. Table 1 shows the implications of partial treatment using data from the SVORI evaluation.^24^ The percent treated for the SVORI and non-SVORI men are shown in columns three and four. Column 2 presents the effect sizes for four interventions as identified by Wanner (2018). If we assume that the recidivism rate without treatment is 20%,^25^ the observed recidivism rate for the SVORI and non-SVORI men as a result of receiving each treatment is shown in columns four and five. Column six shows that the observed differences in recidivism between the two groups in this “thought experiment” are less than two percent—an effect size that would never be detected with typical correctional program evaluations.^26^

**Table 1:** Hypothetical treatment effects with incomplete treatment of the treatment group and partial treatment of the comparison group, assuming untreated recidivism rate is 20 percent

Treatment	Treatment Effect*	Treated (%)		Recidivism Rate		Difference
		SVORI	Non-SVORI	SVORI	Non-SVORI	
Cognitive Behavior Therapy	-0.109	52%	36%	18.87%	19.22%	-1.82%
Substance Abuse Treatment	-0.123	48%	38%	18.95%	19.17%	-1.14%
Vocational Education	-0.167	17%	4%	19.63%	19.91%	-1.42%
General Education	-0.114	53%	43%	18.84%	19.06%	-1.14%

* Estimates from Wanner (2018).

Similar findings emerge when considering the effects on recidivism of interventions such as job training programs that are intended to improve outcomes intermediate to recidivism. Consider the hypothetical impact of a prison job training program on post-release employment and recidivism. The underlying theory of change is that training will increase post-release employment and having a job will reduce recidivism.^27^ Suppose the job training program boosts post-release employment by 30% and that, without the program, 50% of released individuals will find a job. A 30% improvement means that 65% of program participants will find employment. Randomly assigning 100 of 200 individuals to receive the program would result in 50 of those in the control group and 65 of those in the treatment group to find employment. (This outcome assumes everyone in the treatment group receives treatment.) Table 2 shows the treatment effect on recidivism under various assumptions about the impact of employment on recidivism. The table assumes a 50% recidivism rate for the unemployed so, e.g., if the effect of a job is to reduce recidivism by 10% employed individuals will have a recidivism rate of 45%. If there is no effect—i.e., recidivism is independent of being employed—we observe 50% failure for both groups and there is no effect on recidivism rates even if the program is successful at increasing employment by 30%. On the other hand, if being employed eliminates recidivism, no one who is employed will be recidivists and 50% of those unemployed will be recidivists—or 25 of the control group and 17.5 of the treated group. The last column in Table 2 shows the conditional effect of job training on recidivism under the various effects of employment on recidivism shown in column 1. The effects shown in the last column are the same regardless of the assumption about the recidivism rate of the unemployed. So, employment must have a very substantial effect on the recidivism rate to result in a large effect on the observed recidivism rate when, as is reasonable to assume, some members of the control group who didn’t have the training will find employment. As before, this finding underscores the need to carefully consider the mechanism affecting recidivism and potential threats to effect sizes and statistical power.

**Table 2:** Hypothetical effects of job training on employment and recidivism assuming job training increases employment by 30% and control (untreated) employment is 50%

Effect of Job on Recidivism	Recidivism Rates (%)		Number of Recidivists		Effect of Job Training on Recidivism
	Unemployed	Employed	Control	Treated	
-0	50.00%	50.00%	50	50	0.00%
-0.1	50.00%	45.00%	47.5	46.75	-1.58%
-0.2	50.00%	40.00%	45	43.5	-3.33%
-0.3	50.00%	35.00%	42.5	40.25	-5.29%
-0.4	50.00%	30.00%	40	37	-7.50%
-0.5	50.00%	25.00%	37.5	33.75	-10.00%
-0.6	50.00%	20.00%	35	30.5	-12.86%
-0.7	50.00%	15.00%	32.5	27.25	-16.15%
-0.8	50.00%	10.00%	30	24	-20.00%
-0.9	50.00%	5.00%	27.5	20.75	-24.55%
-1	50.00%	0.00%	25	17.5	-30.00%

A third concern is that follow-up periods which typically are 2 years or less may be too short to observe positive impacts of interventions (Lattimore & Visher, 2020). Although this may seem counterintuitive, it is what was observed for the SVORI multisite evaluation. The initial SVORI evaluation focused on the impact of participation with at least 21 months of follow-up following release from prison and showed positive but insignificant differences in rearrests for the SVORI and non-SVORI groups. A subsequent NIJ award provided funding for a long-term (at least 56 months) examination of recidivism for 11 of the 12 adult programs (Visher et al., 2017; also see Lattimore et al., 2012). In contrast to the findings in the original study, participation in SVORI programs was associated with longer times to arrest and fewer arrests after release for both men and women during the extended follow-up period of at least 56 months. Although untestable post hoc, one plausible hypothesis is that the early period following release is chaotic for many individuals leaving prison and failure is likely. Only after the initial “settling out period” are individuals in a position to take advantage of what was learned during program participation. In any event, these findings suggest the need to conduct more, longer-term evaluations of reentry programs.

A final consideration is the indicator of recidivism used to judge the success of a program. Recidivism, which is a return to criminal behavior, is almost never observed. Instead, researchers and practitioners rely on proxies that are measures of justice system indicators that a crime has occurred—arrest, conviction, and incarceration for new offenses—and, for those on supervision, violation of conditions and revocation of supervision. A recent National Academy of Sciences’ publication (2022) highlights some of the limitations of recidivism as a measure of post-release outcomes, arguing that indicators of success and measures that allow for the observation of desisting behavior (defined by the panel as a process—not the sharp break advanced by Bushway) should be used instead. These are valid points but certainly in the short run the funders of interventions and those responsible for public safety are unlikely to be willing to ignore new criminal activity as an outcome.

It is worth highlighting, however, some of the limitations of the binary indicator of any new event that is the usual measure adopted by many researchers (e.g., “any new arrest within x years”) and practitioners (e.g., “return to our Department within 3 years”). These simple measures ignore important dimensions of recidivism. These include type of offense (e.g., violent, property, drug), seriousness of offense (e.g., homicide, felony assault, misdemeanor assault), and frequency of offending (equivalent to time to the recidivism event). As a result, a typical recidivism outcome treats as identical minor acts committed, e.g., 20 months following release, and serious crimes committed immediately. Note too that this binary indicator fails in terms of being able to recognize desisting behavior, that is, where time between events increases or the seriousness of the offense decreases. Survival methods and count or event models address the frequency consideration. Competing hazard models allow one to examine differences between a few categories of offending (e.g., violent, property, drug, other). The only approach that appears to have tackled the seriousness dimension is the work by Sherman and colleagues (Sherman et al., 2016; also, see www.crim.cam.ac.uk/research/thecambridgecrimeharminde) who have developed a Crime Harm Index that is based on potential sentences for non-victimless crimes. To date, statistical methods that can accommodate the three dimensions simultaneously do not, to my knowledge, exist. At a minimum, however, researchers should use the methods that are available to fully explore their recidivism outcomes. Logistic regression models are easy to estimate and the results are easily interpretable. But an intervention may be useful if it increases the time to a new offense or reduces the seriousness of new criminal behavior.

## A Recapitulation

The last forty years or so have seen strides at identifying interventions that are promising, but much work remains to be done to find programs that result in substantial, broad-based improvements. Challenges in program development and implementation, partial treatment of treatment groups *and* control groups, and limited focus on recidivism as a binary indicator of failure were highlighted as some of the issues confronting practitioners and evaluators.^28^ There is reason for optimism—if expectations are realistic from both a programmatic and methodological perspective: Identify promising programs, apply best practices of implementation science, calculate reasonable statistical expectations, and build on what has been tried.

## Conclusions

In the past several decades, dramatic increases in crime resulted in large-scale legislative changes and expenditures. Correctional populations dramatically increased even as crime rates plunged. In addition, despite large increases in funding to law enforcement and other justice agencies, the number of offenses cleared declined. During this time, there were multiple federal initiatives focused on reducing criminal recidivism. Some, such as the Residential Substance Abuse Treatment (RSAT) programs, focused singularly on reducing drug use, while others focused broadly on addressing the multi-faceted needs of justice-involved individuals.

These changes occurred in a context of a highly decentralized approach to criminal justice, one that creates a myriad of costs and incentives. For example, if a federally funded reentry program reduces crime, the immediate agency beneficiaries are local law enforcement (due to fewer crimes to solve), prosecution (due to fewer crimes to prosecute), and the courts (due to fewer cases to try). That can reduce admissions to prison. But for cost-savings to occur, agencies have to respond to reductions in crime by reducing costs. That tends to run counter to the natural inclination of administrators, especially if it means reducing staffing. And it runs counter to what happened as crime declined over the last roughly 30 years.

We increasingly have research evidence that some programs can reduce recidivism, but many challenges, such as underpowered research designs, sometimes undermines this evidence. Even so, it is important to note that even modest reductions in recidivism imply opportunities to avert substantial numbers of crimes and subsequent criminal justice system processing and costs.

This essay suggests that it is time to embrace the modest improvements in recidivism that have been forthcoming from programs that have been subjected to the most rigorous evaluations. And it suggests that it is time to downsize our expectations for a “silver bullet” and, instead, prepare for a long-term and sustained investment in programming that will improve, refine and augment programs and approaches that “work.” By using “what works” today as the basis for the successful adaptation of multi-faceted programs that address the multiplicity of offender needs, criminal justice policy and practice will develop the tools needed to help a heterogeneous population of prisoners successfully reenter their communities.

Finally, as policymakers grapple with a recent increase in violent crime, it is important to recognize that the “tough-on-crime” responses of the twentieth century led to a 252% increase in the number of citizens under legal system control—including a 312% increase in prison populations—between 1980 and 2000. Correctional populations peaked in 2008 but in 2019 remain 255% above 1980 levels with more than 6.5 million individuals in prisons, jails, or on probation or parole.^29^ As the current administration proposes the Safer America Plan, it is important that proper attention be addressed to assure that the result of these expenditures is not to reinvigorate the mass incarceration and mass supervision that followed the adaptation of the as the 1984 Pretrial Reform Act and the Violent Offender Incarceration and Truth-in-Sentencing Act of 1994. And it is important that we attend to widespread support for high-quality implementation of programs that have been shown to reduce recidivism.

## Data Availability

Data are cited from a variety of sources. Much of the BJS data cited are available from the National Archive of Criminal Justice Data, Interuniversity Consortium for Political and Social Research. The SVORI data and the Second Chance Act AORDP data are also available from NACJD.

## Footnotes

1 Some of the ideas presented here were initially explored in Lattimore (2020) and Lattimore et al. (2021).

2 Data 1960 to 1984 are FBI, Uniform Crime Reports, prepared by the National Archive of Criminal Justice Data; downloaded March 5, 2006; data from 1985 to 2020 are from https://crime-data-explorer.app.cloud.gov/pages/explorer/crime/crime-trend, downloaded July 12, 2022.

3 Violent crime commands the most attention and hence is the focus here, but property crimes are much more prevalent—directly affecting many more individuals. Property crime rates also increased in the 1960s and 1970s. The property crime rate increased from 1,726.3 per 100,000 in 1960 to 4,660.2 in 1994—an 170% increase. The property crime rate peaked in 1980 at 5,353.3 per 100,000—a 210% increase over 1960.

4 Data for 1960 and 1970 prisoners are from Cahalan, M.W. and Parsons, L.A. (1986). Data from 1980–2014 are from Glaze, L., Minton, T., & West, H. (Date of version: 12/08/2009) and Kaeble, D., Glaze, L., Tsoutis, A., & Minton, T. (2015). Data from 2015–2020 are from Kluckow, DSW, & Zeng, Z. (Date of version: 3/31/2022).

5 As noted in footnote 3, property crime rates also rose between 1960 and 1980—peaking at 5,353.3 per 100,000. With some minor fluctuations, the property crime rate has declined steadily since the 1980s and was 1958.2 per 100,000 in 2020.

6 The Comprehensive Drug Abuse Prevention and Control Act of 1970 (PL 91–513); the Organized Crime Control Act of 1970 (PL 91–452); the District of Columbia Court Reorganization and Criminal Procedure Act of 1970 (PL 91–358); and the Omnibus Crime Control Act of 1970.

7 https://www.ncjrs.gov/txtfiles/billfs.txt

8 The trend shown in Fig. 9 continued a trend. Between 1982 and 1997, total justice expenditures increased 125% from $84.1 billion to $189.5 billion (2007 dollars), Kyckelhahn, T. (2011).

9 Data are from the FBI Crime in the United States publications for 1980, 1991, 1995, 2000, 2010 and 2019 https://ucr.fbi.gov/crime-in-the-u.s/. Numbers of offenses cleared were estimated by multiplying the offenses known by the offense clearance rates reported by the FBI.

10 https://www.nytimes.com/2022/07/31/opinion/richard-nixon-america-trump.html

11 https://www.whitehouse.gov/briefing-room/statements-releases/2022/08/01/fact-sheet-president-bidens-safer-america-plan-2/

12 For some thoughts on recommendations for reforms for pretrial and sentencing see Lattimore, Spohn, & DeMichele (2021). This volume also has recommendations for reform across the justice system.

13 Safety and Justice Challenge.

14 https://www.arnoldventures.org/

15 https://www.prisonpolicy.org/research/economics_of_incarceration/

16 For an example of how a minor traffic offense can result in thousands of dollars in fines and fees for extensive terms of private probation see In Small-Town Georgia, A Broken Taillight Can Lead to Spiraling Debt—In These Times.

17 See for example, https://www.propublica.org/article/florida-felonies-voter-fraud

18 https://www.arnoldventures.org/work/public-defense

19 https://www.samhsa.gov/gains-center

20 https://www.nami.org/Advocacy/Crisis-Intervention/Crisis-Intervention-Team-(CIT)-Programs

21 https://www.eugene-or.gov/4508/CAHOOTS

22 In 2005, the Uniform Crime Reports reported 1,197,089 known violent offenses and 8,935,714 known property offenses or a ratio of about 1:9. Clearance rates were 45.5% for violent and 16.3% for property crimes known to police. The estimated total number of arrests for 2005 was 14,094, 186. Thus, the violent and property arrests account for about 72% of all arrests. Of course, these estimates rest on many assumptions—in some cases, these assumptions would imply that we are estimating the lower bound, since each member of our study population is allowed only one arrest while many will have many more than one. On the other hand, to the extent that individuals are arrested who have committed no offenses, the estimates would over represent the impact of a reduction in crime. The goal here was not to generate a precise estimate but to illustrate that a 10% reduction in recidivism translates into substantial reductions in crime.

23 A model-based estimate of the effect of non-residential drug treatment on 134,000 drug-involved individuals admitted to probation in Florida showed treatment reduced arrests by more than 20% (Lattimore et al., 2005a, 2005b). This analysis was extended to a cost-effectiveness framework in which it was shown that it would be cost effective to spend $1000 treating all drug-involved probations as long as the average cost of an arrest averted (including arrest, and the costs of judicial processing and corrections) is at least $6,463.

24 This examination of the impact of partial treatment was first presented in Lattimore & Visher (2013). The estimates of treatment effects have been updated from the earlier analysis using the more recent effect size estimates from Wanner (2018). Although the newer effect size estimates are larger than the previous estimates from Drake, Aos, & Miller (2009), the conclusions in terms of impact on recidivism were similar. The calculation for effects on recidivism rates are based on the following calculation. For any group for which some receive treatment and others do not, the recidivism rate for the group is.

$$R=r*(1-T*p)$$

where R = recidivism rate for the group, r = recidivism rate in the absence of treatment, T = percentage of group that is treated, and p = the percentage reduction in recidivism due to treatment (the treatment effect). Differences in outcomes are constant with respect to the assumed recidivism rate in the absence of treatment.

25 Differences in outcomes are constant with respect to the assumed recidivism rate without treatment.

26 Lipsey (1998) discusses the issue of underpowered evaluations.

27 A similar example was presented in Lattimore, Visher, & Steffey (2010).

28 Although not addressed here because of page limitations additional important methodological considerations include whether a comparison group exists for some interventions such as incarceration (see Lattimore & Visher 2021 for a brief discussion) and, even more challenging, whether replication is even possible given the heterogeneity of context and populations. For an interesting consideration of the implications of the latter for examining the impact of incarceration see Mears, Cochran & Cullen (2015).

29 Correctional populations dropped dramatically in 2020 as law enforcement and the criminal justice system adapted to COVID-19.
